# Mind wandering on command

**DOI:** 10.3389/fpsyg.2024.1448226

**Published:** 2024-09-05

**Authors:** Adrian B. Safati, Thomas H. Carr, Cassandra J. Lowe, Daniel Smilek

**Affiliations:** ^1^Department of Psychology, University of Waterloo, Waterloo, ON, Canada; ^2^Department of Psychology, Michigan State University, East Lansing, MI, United States; ^3^Department of Psychology, University of Exeter, Exeter, United Kingdom

**Keywords:** attention, mind-wandering, sustained attention, thought control, executive control, experience sampling

## Abstract

Three experiments (*N* = 336) examined whether participants can systematically adjust levels of mind wandering on command. Participants performed four blocks of the metronome response task (MRT) in which they pressed a spacebar in sync with a steady audio tone. Levels of spontaneous and deliberate mind wandering were measured using intermittent thought probes. Performance was indexed with MRT response time variability and omission errors. Each block started with instructions to mind wander either 20, 40, 60, or 80% of the time. Analysis was primarily conducted using linear mixed effects models. We found that mind wandering (spontaneous and deliberate), response time variability, and omission errors increased progressively with instructions to mind wander more and that these instruction-related changes were larger for deliberate than spontaneous mind wandering (Experiments 1–3). This pattern held regardless of whether participants’ eyes were open or shut (Experiment 2). Relative to a control group receiving no commands to mind wander, instructing people to mind wander 60 or 80% of the time led to more deliberate mind wandering, and strikingly, asking people to mind wander 20% of the time led to less spontaneous mind wandering (Experiment 3). Our results suggest that individuals can titrate mind wandering experiences to roughly match instructed levels indicating that mind wandering can be manipulated through simple instructions. However, other features of the data suggest that such titration is effortful and may come with a cost to performance.

## 1 Introduction

Our ability to direct our attention is often examined in contexts in which we are expected to manage our wandering thoughts and focus on the details of our environment and the tasks to be performed within it. Studies have shown that under such conditions, our thoughts can wander from a primary task in varying amounts, with the amount depending on a multitude of factors, including momentary task demands (Seli et al., 2018c), time on task (Farley et al., 2013; Thomson et al., 2014; Krimsky et al., 2017; Brosowsky et al., 2020), interest in the stimuli (Smallwood et al., 2009b; Kane et al., 2017), boredom (Danckert et al., 2018; Blondé et al., 2022), mood (Smallwood et al., 2009a), levels of motivation (Seli et al., 2019), drug use (Sayette et al., 2009), a person’s physiological state such as sleep deprivation (Poh et al., 2016) and sleepiness (Stawarczyk and D’Argembeau, 2016), and individual characteristics such as age (Maillet and Schacter, 2016), attention-traits (Pereira et al., 2020), ADHD symptomology (Seli et al., 2015b), and working memory capacity (Robison and Unsworth, 2018). What remains relatively unexplored, however, is how much control people have over their levels of off-task thought – i.e., their “mind wandering” (Seli et al., 2018b; Smallwood and Schooler, 2006; Turnbull et al., 2019). In the experiments presented here we explore whether participants can regulate their levels of mind wandering deliberately based on instructions to mind wander a specific amount.

Mind wandering has become a focus of research partly because it has been linked to impaired task performance in many contexts. In everyday life, mind wandering has been associated with unsavory outcomes such as lower academic achievement (Seli et al., 2016c; Wammes et al., 2016; Pereira et al., 2020), poor driving (He et al., 2011; Yanko and Spalek, 2014; Qu et al., 2015), or medical errors (Smallwood et al., 2011b). Mind wandering-related performance costs have also been documented in many laboratory tasks (see review by Mooneyham and Schooler, 2013). One example involves a task that requires participants to press a button in synchrony with the sound of a metronome (Seli et al., 2013b). While completing this metronome response task (MRT), participants are intermittently presented with experience sampling probes, asking them to report on their level of mind wandering. In this task, performance is indexed primarily by the trial-to-trial variance in the time between the onset of the tone and the response for a given trial (i.e., rhythmic response time variance: RRTv), and secondarily by the proportion of trials on which participants failed to respond to a tone (i.e., omission errors). Across multiple studies it has been shown that increased mind wandering during the MRT is related to more trial-to-trial response time variance (i.e., higher RRTv; Anderson et al., 2021; Seli et al., 2013b) and sometimes higher omissions (e.g., Seli et al., 2019), both of which indicate impaired performance.

Why does mind wandering often lead to poor performance on assigned tasks? One explanation is mind wandering requires the same limited attentional resources needed to effectively carry out attention-demanding tasks (see Smallwood and Schooler, 2006). On this “limited-resource” view, when attentional resources are misallocated to mind wandering, fewer resources are available for the task at hand, which impairs performance if the task is attentionally demanding. Mind wandering has also been construed as a failure of executive control (McVay and Kane, 2010; Kane and McVay, 2012; Thomson et al., 2015). The “control-failure” view (McVay and Kane, 2012) is consistent with studies showing that mind wandering increases with decreases in cognitive control. States associated with physiological impairments, known to decrease control, like alcohol intoxication (Sayette et al., 2009) or sleep deprivation (Poh et al., 2016), which are associated with increased mind wandering and a breakdown of the ability to adaptively regulate mind wandering to preserve performance in response to changes in task demand (Poh et al., 2016). The control-failure view complements the limited-resource account in that executive control can be understood as the process governing the allocation of the limited pool of attentional resources (Smallwood, 2013; Thomson et al., 2015). Accordingly, if it is assumed participants are diligently attempting to allocate all their resources to the primary task at hand (e.g., the one assigned to them by the experimenter in the laboratory), then any diversion of resources to mind wandering would be the result of a failure of control. Critically, from this perspective mind wandering is considered an unintentional (i.e., spontaneous) event, which was the dominant position in the recent resurgence of mind wandering research (Christoff et al., 2016; literature review in McVay and Kane, 2010).

However, studies have shown during various tasks, participants report they mind wandered not only spontaneously, but also *deliberately* (Seli et al., 2015a, 2016a, 2019; Wammes et al., 2016). For example, Seli et al. (2019) had participants complete the MRT in which they pressed a spacebar in sync with a regularly occurring audio tone and were intermittently probed to report whether they were on task, mind wandering intentionally, or mind wandering unintentionally. The results showed participants reported mind wandering both unintentionally and intentionally, with *intentional* mind wandering occurring roughly 20% of the time. Furthermore, Seli et al. (2019) found that as intentional mind wandering increased, omissions on the MRT also increased. Along similar lines, (Robison and Unsworth, 2018) showed people report experiencing both unintentional and intentional episodes of mind wandering while performing cognitive tasks designed to strain working memory. Structural equation modeling of their data revealed spontaneous mind wandering was primarily predicted by individuals’ working memory capacity as indexed by task performance and their levels of self-reported alertness, while deliberate mind wandering was related mainly to participants’ levels of motivation to perform well on the task. When taken together, these and other studies (Seli et al., 2015a, 2016b; Zhang et al., 2021) suggest (1) that people mind wander both spontaneously and deliberately, (2) that spontaneous and deliberate mind wandering are related but also distinct and are associated with and/or driven by different factors, and (3) that both types of mind wandering can impact performance.

We turn now to the question addressed by our experiments: How much control do people have over their mind wandering? Reports of intentional mind wandering suggest the possibility that people could regulate their levels of mind wandering at will to match an instructed amount. Support for the notion that people can allocate their attentional resources based on instructions to prioritize specific content comes from earlier studies of attention (e.g., Broadbent, 1952; Moray, 1959; Sperling and Melchner, 1978; see also Pillsbury, 1908). For example, in studies reported by (Sperling and Melchner, 1978) participants were briefly shown visual search displays (to eliminate the role of eye saccades) consisting of letters and numbers arranged in an inner square and an outer square. Participants were required to detect and report two target items present in the displays. Critically, in separate blocks of trials participants were instructed to pay attention to (1) the items in the outer square, or (2) the items in the inner square, or (3) the items in both inner and outer squares equally. The results showed participants were able to follow the instructions and when instructed to pay attention to either the inner or outer squares their detection of the target in that (attended) square improved, but this came at the expense of detecting targets in the other (unattended) square. These results are consistent with the view that attention is a limited capacity resource and people can control the allocation of that resource to favor some information at the expense of other information (see Tombu and Jolicœur (2003) for a discussion of capacity sharing models). Based on these findings it seems conceivable that people could similarly control their allocation of attention to external task requirements or to mind wandering based on instructions.

While to date most experimental manipulations of mind wandering have varied mind wandering indirectly (e.g., by manipulating resource demands; Brosowsky et al., 2021; Poh et al., 2016; Seli et al., 2018c; Smallwood et al., 2011a; Thomson et al., 2013), one study has explored whether people can directly control their levels of mind wandering based on instructions (Kawagoe et al., 2018). Kawagoe et al. (2018) measured resting state differences in functional connectivity following instructions to either “think of nothing” or “let your mind wander.” The results showed asking participants to let their minds wander elicited characterizable differences in both reports of mind wandering and the activity of their default mode network, a series of brain structures associated with internally-directed thought and self-reflection, which has been associated with mind wandering (Christoff et al., 2016). While these results suggest that instructions can effectively elicit differences in mind wandering, the self-report measures employed by Kawagoe et al. (2018) did not distinguish between mind wandering subtypes (i.e., deliberate versus spontaneous) and their instructions were binary (i.e., instructing participants to either “try to think of nothing” or to “let your mind wander”). Thus, it remains to be determined, both the degree of intentional control that can be achieved and whether the exercise of such control impacts spontaneous as well as deliberate mind wandering.

Extending this prior work, here we examined the ability of individuals to regulate their mind wandering to match instructed levels, specifically focusing on whether instructions to mind wander specific amounts would influence deliberate and spontaneous mind wandering differently, and whether it would influence task performance. Developing a deeper understanding of the extent to which people can mind wander on command would be useful because it would allow researchers to manipulate mind wandering directly while holding other contextual factors (e.g., task demands, motivation, cognitive abilities, individual characteristics) constant. Studying the mechanisms involved when people are trying to mind wander while managing its place in overall performance may also complement our understanding of what happens when people are supposed to be avoiding mind wandering, which has been the dominant focus in prior investigations. It may also provide further evidence that we can flexibly adjust mind wandering levels depending on task demands, as suggested in Rummel and Boywitt (2014) for instance.

To address these issues, in three experiments we had participants perform four blocks of the MRT with instructions to mind wander different amounts. We chose the MRT as the primary task because (1) it is an auditory task, which prevents participants from disengaging from the task simply by closing their eyes (as they could with a visual task), (2) it yields levels of intentional and unintentional mind wandering that are within an optimal range for measurement (i.e., prior work demonstrates a baseline of 42% for spontaneous and 26% for deliberate mind wandering which were reduced to 33 and 16%, respectively, in a motivation manipulation (Seli et al., 2019), suggesting that reports of mind wandering during the task could be increased or decreased without scale attenuation from ceiling or floor effects), and (3) because it provides performance metrics that have previously been linked to variations in levels of mind wandering (Seli et al., 2013b; Anderson et al., 2021). At the start of each block of the MRT participants were given instructions to mind wander either 20, 40, 60, or 80% of the time. We assessed both deliberate and spontaneous mind wandering throughout the task using thought probes and we tracked task performance in terms of response time variability and omissions as has been done before.

When making predictions about the primary outcomes of the present studies, it is important to keep in mind that instructing participants to mind wander a certain amount effectively creates a situation of triple-tasking, in which central limited resources have to be shared between (1) the primary task (i.e., the MRT), (2) mind wandering, and (3) keeping track of one’s level of mind wandering (i.e., metacognition; Schooler, 2002; Schooler et al., 2011; Seli et al., 2017). This consideration is particularly pertinent when it comes to predictions about *spontaneous* mind wandering. On the one hand, spontaneous mind wandering might *increase* with instructions to mind wander more because such instructions might engender a level of control that entails less inhibition and is more permissive of spontaneous mind wandering, as might be the case with reduced levels of motivation (see Seli et al., 2019). On the other hand, spontaneous mind wandering might *decrease* with instructions to mind wander more because such instructions might lead to greater demands on metacognitive functions and thus task load, which is known to decrease spontaneous mind wandering. The predictions about *deliberate* mind wandering are less complex. If participants can regulate their levels of mind wandering on demand, then we would expect deliberate mind wandering reports to increase with increases in instructed levels of mind wandering, since this sort of mind wandering is ostensibly under conscious control. In addition, because increased mind wandering is associated with poorer performance on the MRT (Seli et al., 2013b), we expected that MRT response time variability and omissions will increase as participants are instructed to mind wander more.

## 2 Experiment 1

Experiment 1 had several aims. First, as noted above, we examined whether people can regulate their levels of mind wandering in a graded fashion when asked to mind wander different amounts, and whether the impact of instructed mind wandering levels differed between spontaneous and deliberate reports of mind wandering. To this end, participants were instructed to mind wander either 20, 40, 60, or 80% in different blocks of the MRT. Participants were randomly assigned to one of three groups. In the Counterbalanced group participants received all possible permutations of the orders of the 20, 40, 60, and 80% mind wandering instruction across blocks. The Ascending group received the instructed levels of mind wandering in increasing order (i.e., 20, 40, 60, 80). Finally, the Descending group received the levels of mind wandering instructions in a decreasing order (i.e., 80, 60, 40, 20). We intermittently measured the degrees of both deliberate and spontaneous mind wandering using separate sliding scales and we also monitored performance in the form of response time variability and omission errors. Our expected outcomes for this counterbalanced condition were described above.

In addition, we examined how the mind wandering instructions would interact with natural trends in mind wandering that occur from time spent on a task. Accordingly, we included one group of participants (the Ascending instructions group) who received mind wandering instructions in ascending order across successive blocks (i.e., 20%, then 40%, then 60% and finally 80%). This order of instructed mind wandering should be consistent with and perhaps facilitate the tendency to mind wander more over time. In contrast, another group of participants (the Descending instruction group) were instructed to mind wander in decreasing fashion across successive blocks (i.e., starting with 80%, then 60, 40, and 20%). We expected that this ordering of instructed levels of mind wandering should counteract the natural trend of increased mind wandering over time on task. We hypothesized that the Ascending group would exhibit larger changes across instruction blocks in their reports of mind wandering, rhythmic response time variability, and omissions in the MRT than the Descending group.

Finally, in Experiment 1 we also took the opportunity to replicate several trends previously observed in the literature. One such pattern mentioned above is that mind wandering increases with time on task (Farley et al., 2013; Thomson et al., 2014; Krimsky et al., 2017; Brosowsky et al., 2020) which we sought to now replicate by considering just the counterbalanced condition. Another pattern concerns how mind wandering changes as a function of the interval between thought probes. Prior studies have shown as the interval between thought probes increases, so do reports of overall mind wandering (Seli et al., 2013a). We expected to find similar results in our entire sample of data. Still another pattern involves the relation between mind wandering reports and performance on the preceding trials (Seli et al., 2013b; Anderson et al., 2021). Researchers have focused on this relation because the preceding trials likely best reflect the behavior associated with the mental state reported for that specific probe. We analyzed both MRT response time variability and omissions in the set of trials preceding thought probes in all groups, expecting to find more response time variability and omissions would be associated with subsequently larger reports of mind wandering.

### 2.1 Methods

Our research was approved by the Office of Research Ethics at the University of Waterloo. The deidentified experimental data and analysis code for the three experiments are available at: https://osf.io/sn28j/?view_only=d33caa24d1dd46fc96d1b31abacc2914. Study materials are available upon request.

#### 2.1.1 Participants

Three groups of 48 participants (*N* = 144) were recruited from the University of Waterloo undergraduate SONA research pool to participate in a 30-min study. The group size of 48 was selected to allow each permutation of the 20, 40, 60, 80% instruction order to be run twice in the Counterbalanced group. Two participants were replaced, one due to non-compliance with instructions, another due to an error in the instruction delivery by a research assistant. Data collection occurred in person with participants receiving course credit as remuneration. The sample consisted of 108 Females, 34 Males, and two participants who did not disclose their sex, with ages ranging from 17 to 49 (*M* = 20.1, *SD* = 3.9).

#### 2.1.2 Materials

*Metronome response task (MRT)*: In the MRT participants are presented with a rhythmic auditory tone simulating a metronome and are instructed to press the space bar in synchrony with the tone. The instructions specified participants should try to time their responses to match the start of the tone (i.e., anticipating the tone, not just reacting after it). Our implementation of the MRT consisted of a practice block of 16 trials followed by 4 experimental blocks of 224 trials (896 trials total). Each trial was 1,300 ms long consisting of 650 ms of silence followed by the 75 ms tone, and another 575 ms of silence. The MRT was intermittently interrupted with a screen presenting the mind wandering thought probes; the task resumed once the responses to the probe were provided. Each experimental block of the MRT lasted approximately 5½ minutes, varying slightly by how quickly participants completed the probes.

Performance during the MRT was assessed by examining rhythmic response time variability, and omissions (Seli et al., 2013b). Specifically, rhythmic response times were calculated from the time difference between participants pressing the spacebar and the onset of the metronome tone. In line with prior analysis of the MRT (Seli et al., 2013b) we calculated rhythmic response time variability (RRTv) from the variance in a rolling window of 5 trials. Missing responses were filtered to ensure that each window consisted of 5 trials. The variances derived from these 5-trial windows were averaged to generate the RRTv. As with prior analyses of the MRT, a natural-log transformation was applied to the RRTv to help normalize the positive skewness in the data. Omissions were observed in trials where the participant failed to press the space bar, excluding those at the start of a block or immediately following a thought probe.

*Mind wandering thought probes*: Participants reported on their levels of deliberate and spontaneous mind wandering on a single screen that interrupted the MRT. They were instructed to answer the questions based on their experience before the probe. One question asked, “How much were you deliberately mind wandering?” and the other question asked, “How much were you spontaneously mind wandering?” Participants responded by clicking then dragging a slider on continuous scales located below each question. The end of each slider was anchored with “*Not at all*” on the left and “*All the time*” on the right. Participants were not prevented from reporting high levels of both deliberate and spontaneous mind wandering simultaneously. The presentation order of the deliberate and spontaneous mind wandering scales was randomized between participants. The slider scale included a vertical line demarcating each of the ends of the slider, as well as three other equally spaced vertical lines to serve as general landmarks. To avoid introducing an anchoring bias the response slider does not appear until after participants have clicked a point on the scale.

Participants had an opportunity to practice answering a probe during the practice block of the MRT. During each of the four experimental blocks there were four thought probes distributed throughout, with the probes appearing pseudo randomly after at least five trials of each quarter block of 56 trials. This ensured a minimum separation of at least five trials and that probes could appear up to 107 trials apart. The wide range of intervals enabled better testing of the notion that longer intervals produce more mind wandering.

Throughout the experiment each participant completed a total of 16 probe reports in addition to the one in the practice block. The jittered distribution of the probes was done to prevent anticipatory responses that can happen when events occur at fixed intervals (Seli et al., 2018a).

#### 2.1.3 Procedure

Upon entering the testing room, participants were seated in front of a computer and given the study instructions. Participants were informed the study was examining the extent to which people can consciously regulate their levels of mind wandering during a task. The concept of mind wandering was introduced to participants as a common experience in daily life when thoughts drift from one’s current task and immediate external environment to an internal stream of consciousness. Participants were provided with examples of what mind wandering could entail. Examples included reading a book, going for a walk, or taking a shower and instead of staying focused on the current task and environment individuals can find their thoughts drifting to the T.V. show they watched last night, their plans for the weekend, or what it means to be happy. To ensure clarity in our instructions participants were given examples of mentation that we do not consider to be mind wandering such as performing mental arithmetic or counting objects in their environment. While counting objects and performing arithmetic could be means of attentional disengagement from the MRT, they could be construed as alternative tasks that impose constraints on the freer movement of thoughts that is typical, even definitional, of mind *wandering* (Christoff et al., 2016; Mills et al., 2021).

Our definition of mind wandering for participants was then extended to include the concepts of spontaneous and deliberate mind wandering. We used an example of a university lecture to draw the distinction between the two subtypes. We illustrated how spontaneous mind wandering was like the experience of sitting in a class and trying to focus but finding that despite one’s intentions to be attentive their thoughts start drifting away as they begin to mind wander unintentionally. For deliberate mind wandering we used the analogy of trying to mentally escape from a tedious lecture. Instead of trying to be attentive, here one is trying to tune out the speaker in an intentional attempt to let their mind wander somewhere else. We clarified that throughout the experiment participants could engage in both deliberate and spontaneous mind wandering and when asked to report on their experiences there could be overlap between the two.

Participants then completed 4 blocks of the MRT, receiving instructions to mind wander either 20, 40, 60, or 80% of the time at the start of each block. They were instructed to do their best to adjust their mind wandering to the instructed amount. Participants completed four thought probes throughout each block. For these thought probes participants were instructed to report their mind wandering “right before the probe appeared,” then to continue with the block still trying to mind wander in accord with the same instructed amount.

Participants were tested one at a time in a laboratory testing room, and the MRT and thought probes were presented using version 2022.2 of PsychoPy (Peirce et al., 2019). Some participants consented to being recorded; we intend to analyze postural changes in these recordings in a future study in which we are planning to characterize the embodiment of attentional states. There was a minor coding bug that resulted in the first participant of the experiment receiving an extra probe in their second block. The error was corrected for all other participants and that participant’s data was still included in the analysis.

### 2.2 Analysis

We used a series of independent theoretically informed models to conduct statistical tests of our hypotheses. Model summaries, including the formulas, the observations examined, performance indicators, and ANOVAs for the predictors are available in Tables 1, 2.

**Table 1:** ANOVA tables for the linear mixed effects models examining mind wandering reports and response time variability in Experiment 1.

Model	Parameter	*SS*	*MS*	*df_Num_*	*df_Den_*	*F*	*p*
Model 1.1	Model Formula: MW ~ instruction*type + probe_interval*type + time*type + (instruction | participant:type) Model Data: Counterbalanced Group, Observations = 1,535, Performance: R^2^_Marginal_ = 0.199, R^2^_Conditional_ = 0.621						
	Instruction	23947.83	7982.61	3	91.8	28.085	<0.000
	Type	13927.48	13927.48	1	290.3	49.001	<0.000
	Probe_interval	12544.02	12544.02	1	1226.0	44.134	<0.000
	Time	235.41	235.41	1	283.4	0.828	0.364
	Instruction:type	6065.42	2021.81	3	91.8	7.113	<0.000
	Type:probe_interval	11392.44	11392.44	1	1226.0	40.082	<0.000
	Type:time	21558.15	21558.15	1	283.4	75.848	<0.000
Model 1.2	Model Formula: RRTv ~ instruction + (instruction | participant) Model Data: Counterbalanced Group, Observations = 40,927, Performance: R^2^_Marginal_ = 0.006, R^2^_Conditional_ = 0.376						
	Instruction	21.96	7.32	3	46.7	6.08	0.001
Model 1.4	Model Formula: MW ~ instruction*type*group + probe_interval*type + (instruction | participant:type) Model Data: Ascending and Descending Groups, Observations = 3,065, Performance: R^2^_Marginal_ = 0.221, R^2^_Conditional_ = 0.667						
	Instruction	55892.61	18630.87	3	187.9	73.944	<0.000
	Type	8939.31	8939.31	1	301.4	35.479	<0.000
	Group	1880.49	1880.49	1	187.8	7.464	0.007
	Probe_interval	26678.71	26678.71	1	2346.7	105.886	<0.000
	Instruction:type	14194.51	4731.50	3	187.9	18.779	<0.000
	Instruction:group	2918.78	972.93	3	187.9	3.861	0.010
	Type:group	301.28	301.28	1	187.8	1.196	0.276
	Type:probe_interval	41386.10	41386.10	1	2346.7	164.258	<0.000
	Instruction:type:group	7945.45	2648.48	3	187.9	10.512	<0.000
Model 1.5	Model Formula: RRTv ~ instruction*group + (instruction | participant) Model Data: Ascending and Descending Groups, Observations = 82,240, Performance: R^2^_Marginal_ = 0.010, R^2^_Conditional_ = 0.372						
	Instruction	28.90	9.63	3	93.4	7.428	<0.000
	Group	0.02	0.02	1	94.0	0.014	0.906
	Instruction:group	36.43	12.14	3	93.4	9.365	<0.000
Model 1.7	Model Formula: MW ~ RRTv*type + probe_interval*type + time*type + (1 | participant:type) Model Data: All Groups, Observations = 4,580, Performance: R^2^_Marginal_ = 0.095, R^2^_Conditional_ = 0.404						
	RRTv	49839.520	49839.520	1	3166.564	108.525	<0.000
	Type	146.012	146.012	1	2874.091	0.318	0.573
	Probe_interval	49236.571	49236.571	1	4290.233	107.212	<0.000
	Time	14918.298	14918.298	1	4331.666	32.484	<0.000
	RRTv:type	2224.754	2224.754	1	3166.564	4.844	0.028
	Type:probe_interval	39694.336	39694.336	1	4290.233	86.434	<0.000
	Type:time	81813.073	81813.073	1	4331.666	178.147	<0.000
Model 1.8	Model Formula: MW ~ omissions*type + probe_interval*type + time*type + (1 | participant:type) Model Data: All Groups, Observations = 4,592, Performance: R^2^_Marginal_ = 0.063, R^2^_Conditional_ = 0.367						
	Misses	15158.636	15158.636	1	4577.808	32.250	<0.000
	Type	51237.558	51237.558	1	884.866	109.007	<0.000
	Probe_interval	35107.943	35107.943	1	4328.516	74.691	<0.000
	Time	9194.806	9194.806	1	4307.331	19.562	<0.000
	Misses:type	238.359	238.359	1	4577.808	0.507	0.476
	Type:probe_interval	39405.557	39405.557	1	4328.516	83.835	<0.000
	Type:time	79171.763	79171.763	1	4307.331	168.436	<0.000

**Table 2:** ANOVA tables for the generalized mixed effects models examining omission errors in Experiment 1.

Model	Parameter	npar	*SS*	*MS*	*F*	*p*
Model 1.3	Model Formula: omissions ~ instruction + (1 | participant) Model Data: Counterbalanced Group, Observations = 41,472, Performance: R^2^_Marginal_ = 0.066, R^2^_Conditional_ = 0.552					
	Instruction	3	206.03	68.68	68.678	<0.000
Model 1.6	Model Formula: omissions ~ instruction*group + (1 | participant) Model Data: Ascending and Descending Groups, Observations = 82,963, Performance: R^2^_Marginal_ = 0.040, R^2^_Conditional_ = 0.577					
	Instruction	3	122.50	40.83	40.833	<0.000
	Group	1	1.18	1.18	1.184	0.039
	Instruction:group	3	205.58	68.53	68.526	<0.000

The dependent measures included reports of spontaneous and deliberate mind wandering (Models 1.1, 1.4, 1.7, 1.8), MRT RRTv (Models 1.2, 1.5), and the odds of omissions (Models 1.3, 1.6). These outcomes were analyzed using the lme4 package (Bates et al., 2015) in R version 4.3.3 (R Core Team, 2024) with linear mixed effects models (Models 1.1, 1.2, 1.4, 1.5, 1.7, 1.8) and generalized mixed effects models using a logit link (Models 1.3, 1.6). Model assumptions were checked using the performance package (Lüdecke et al., 2021). Post-hoc analyses were performed using the emmeans package with Tukey’s HSD to adjust for multiple pairwise comparisons (Lenth, 2023). The linear mixed effects model ANOVA results presented in Table 1 tested significance using the Satterthwaite approximation for degrees of freedom with the lmerTest package (Kuznetsova et al., 2017). The generalized mixed effects model ANOVA results presented in Table 2 used a Likelihood Ratio Test (LRT) to test the significance of the predictors.

In Experiment 1 and subsequent experiments model performance in the tables is indicated by the R^2^_Marginal_ and R^2^_Conditional_ scores. The R^2^_Marginal_ describes the proportion of variance explained by the fixed factors alone, while the R^2^_Conditional_ describes the proportion of variance explained by both the fixed and random factors (Nakagawa et al., 2017). The change between the R^2^_Marginal_ and the R^2^_Conditional_ scores in our models represents the proportion of variance explained by considering inter-individual differences. In models where there is a large degree of between subject variability (e.g., when examining RT variability) we can expect R^2^_Marginal_ scores to be quite low. Post-hoc power analysis of all the experimental models can be conducted using the SIMR package in R (Green and MacLeod, 2016). The code to run this analysis has been made available as part of our OSF repository.

Model 1.1 (Table 1) used data from the Counterbalanced group to examine how our mind wandering instructions (“20,” “40,” “60” or “80”) influenced reports of mind wandering, considering differences in the type of mind wandering report (“spontaneous” or “deliberate”). In the fixed effects we included factors that have been demonstrated to influence reports of mind wandering, i.e., time (the seconds spent on the task), and probe interval (the number of trials preceding the probes). Our random effects specified random intercepts and slopes for each participant, as individuals have different baselines for their mind wandering, and we can expect variations in the responsiveness to our instruction. We considered separate slopes and intercepts for spontaneous and deliberate mind wandering reports as these are distinct constructs.

Model 1.2 (Table 1) used data from the Counterbalanced group to examine how our mind wandering instructions influenced performance on the MRT as measured by rhythmic response time variability scores. The fixed effects component is simpler as the outcome measure is RRTv, instead of mind wandering reports. For the random effects we considered random slopes and intercepts for each participant. As with mind wandering, we expect individuals will have different baselines for their task performance, and we expect individual differences in the responsiveness to our instructions.

Model 1.3 (Table 2) used data from the Counterbalanced group to examine how our mind wandering instructions influenced the odds of omissions in the trials. Our random effects specified only random intercepts to promote convergence of the generalized mixed effects model.

Model 1.4 (Table 1) used data from the Ascending and Descending groups to examine the interaction between our mind wandering instructions, the type of mind wandering report and the group (“Ascending” or “Descending”). The fixed effects again include probe interval; time on task was excluded as much of the variability from the effects of time will be captured by the group x instruction interaction as the instructions are sequenced to produce an interaction with time. The random effects again included random intercepts and slopes, separated for the spontaneous and deliberate mind wandering reports.

Model 1.5 (Table 1) used data from the Ascending and Descending groups to examine how the sequence of our mind wandering instructions in these groups interacts to influence RRTv. For the random effects we again considered random slopes and intercepts for each participant.

Model 1.6 (Table 2) used data from the Ascending and Descending groups to examine how the sequence of our mind wandering instructions in these groups interacts to influence the odds of omissions in the trials. As in Model 1.3 only random intercepts were used to promote model convergence.

Model 1.7 (Table 1) used data from all three groups to examine whether rhythmic response time variability scores predict subsequent mind wandering reports. RRTv was modeled separately from mind wandering instructions as we expect collinearity between these two terms. Here we considered whether the relationship between RRTv and mind wandering reports differed for spontaneous and deliberate mind wandering. Just as in Model 1.1 we included time and probe interval to help explain variability in the outcomes. The random effects included random intercepts for each participant, separated for spontaneous and deliberate reports. There are no random slopes as the RRTv is a continuous measure.

Model 1.8 (Table 1) used data from all three groups to examine whether missing responses predict subsequent mind wandering reports. Here we considered whether the relationship between omissions and mind wandering reports differed for spontaneous and deliberate mind wandering. Again we included time and probe interval to help explain variability in the outcomes. Fixed effects included time and probe interval, but not instructions due to expected collinearity. The random effects included random intercepts for each participant, separated for spontaneous and deliberate reports. Again, there are no random slopes as the number of missing responses is a continuous measure.

### 2.3 Results and discussion

#### 2.3.1 Mind wandering on command in the counterbalanced group

**Figure 1:**
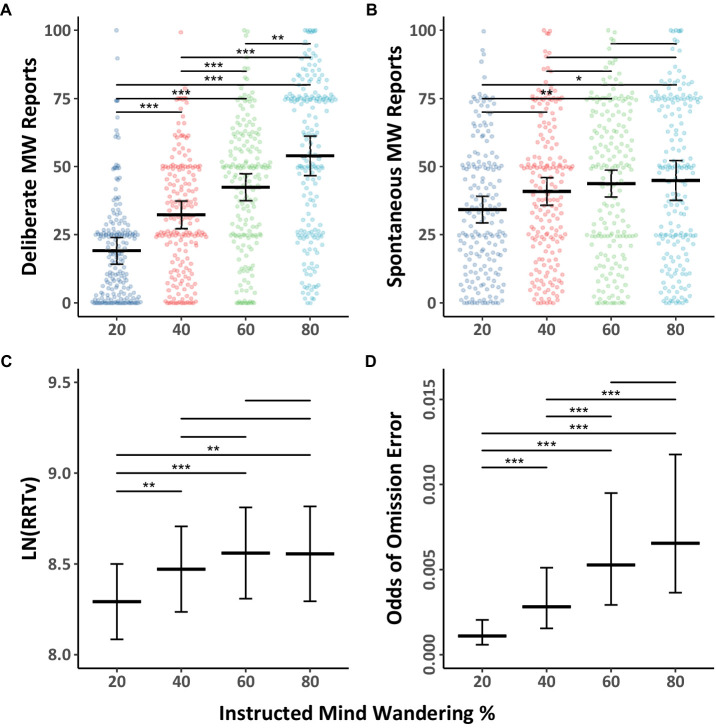
Mind wandering outcomes and MRT performance in the counterbalanced group by level of instructed mind wandering for Experiment 1. The colored points illustrate raw participant responses to the mind wandering probes at every level of mind wandering instruction. The estimated marginal means, and 95% confidence intervals from Model 1.1 for panels **(A,B)**, Model 1.2 for panel **(C)**, Model 1.3 for panel **(D)** are presented in black. Pairwise comparisons with significance scores are shown at the top, Tukey’s HSD was used to adjust for multiple comparisons. The rhythmic response time variability scores presented in panel **(C)** were transformed by applying a natural logarithm function (*p < 0.05, **p < 0.01, ***p < 0.001).

We first addressed our primary aim, which was to explore whether participants can regulate their level of mind wandering when asked to mind wander different amounts, with a focus on both spontaneous and deliberate mind wandering reports in the Counterbalanced group. The mind wandering data were fitted with Model 1.1 (see Table 1) and the data, together with Tukey HSD comparison outcomes across levels of instructed mind wandering, are shown in Figures 1A,B. As can be seen in the figure, the results demonstrate that as participants were instructed to mind wander more, their reports of deliberate and spontaneous mind wandering increased. The model also revealed an instruction by mind wandering type interaction, indicating that there was a greater instruction-related increase in deliberate mind wandering than spontaneous mind wandering.

The behavioral performance data during the MRT as a function of instructed mind wandering levels in the Counterbalanced group are shown in Figures 1C,D together with Tukey HSD comparison results. The RRTv data was analyzed using Model 1.2 (Table 1), while the omission data was analyzed using Model 1.3 (Table 2). The results showed as instructed mind wandering levels increased, performance on the task decreased, as evidenced by increasing rhythmic response time variability score (i.e., higher RRTv), and an increased odds of omission errors. Back transformation of the rhythmic response time variability scores reveals when participants are asked to mind wander 80% of the time their response time variability is 31% higher than when they are asked to mind wander 20% of the time. While the relative odds of omissions were almost six times greater when participants were asked to mind wander 80% of the time compared to 20% of the time, the odds of omissions were quite low in all blocks.

#### 2.3.2 Mind wandering on command in the ascending and descending groups

**Figure 2:**
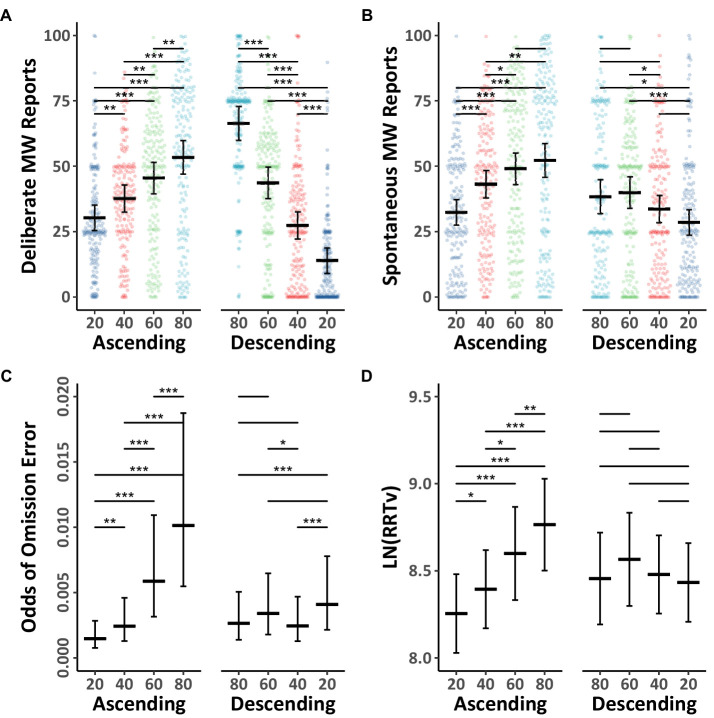
Mind wandering outcomes and MRT performance in the ascending and descending mind wandering instruction groups for Experiment 1. The colored points illustrate raw participant responses to the mind wandering probes at every level of mind wandering instruction. The estimated marginal means, and 95% confidence intervals from Model 1.4 for panels **(A,B)**, Model 1.5 for panel **(C)**, and Model 1.6 for panel **(D)** are presented in black. Pairwise comparisons with significance scores are shown at the top, Tukey’s HSD was used to adjust for multiple comparisons (*p < 0.05, **p < 0.01, ***p < 0.001).

Next, we examined the influence of instructed levels of mind wandering on spontaneous and deliberate probe responses in the Ascending and Descending groups. The mind wandering reports were analyzed using Model 1.4 (see Table 1), and the data, together with Tukey HSD comparisons, are shown in Figures 2A,B. The results showed as participants were instructed to mind wander progressively more in the Ascending group, their reports of both deliberate and spontaneous mind wandering increased. Likewise, as participants were instructed to mind wander progressively less in the Descending group their reports of both deliberate and spontaneous mind wandering decreased. However, in the Descending group, the drop in deliberate mind wandering reports was much larger than the corresponding drop in spontaneous mind wandering. This discrepancy in instruction related changes across the two types of mind wandering were larger in the Descending group than the Ascending group, which led to the instruction by type by group interaction observed in Model 1.4. Thus, when it comes to spontaneous mind wandering, instructing people to mind wander in increasing amounts over time on task appears to interact with natural tendencies to mind wandering more over time on task.

The behavioral outcomes in the Ascending and Descending groups are shown in Figures 2C,D, the data were analyzed using Models 1.5 (Table 1) and 1.6 (see Table 2). As can be seen in the figure, participants in the Ascending group demonstrated a 61% increase in their rhythmic response time variability scores, and a 6.89 times increase in their chances of making omission errors from when they were instructed to mind wander 20% of the time in the first block, to when they were instructed to mind wander 80% of the time in the final block. In contrast, participants in the Descending group demonstrated no significant change in their response time variability scores across any of their blocks and a much smaller increase in their chances of making omission errors. This led to significant instruction by group interactions in Models 1.5 and 1.6. Thus, contrary to the usual decline in performance seen during prolonged task engagement (and which was found in the Counterbalanced and Ascending conditions), participants in the Descending group demonstrated a consistent level of RRTv performance in their responses for approximately 24 min in response to our instructions. While the odds of making omission errors did significantly increase from the first block to the final block in the Descending group, the 0.0041 odds of making an omission error in the final block remained very low relative to the increases seen in the other conditions.

This finding that the mind wandering instructions influence not only mind wandering reports, but also objective performance suggests that the instructed changes in mind wandering reports reflect actual changes in attention to the primary task, which are affecting performance.

#### 2.3.3 Additional observations

**Figure 3:**
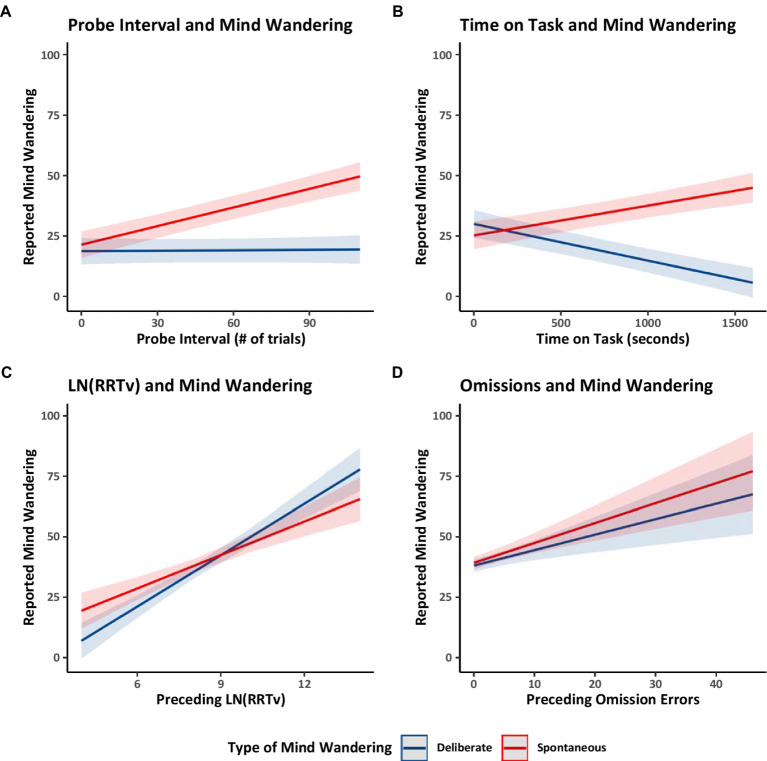
Estimated marginal trends in spontaneous and deliberate mind wandering reports for Experiment 1. Estimated marginal trends for the effects of **(A)** probe interval, **(B)** time on task, **(C)** rhythmic response time variability, and **(D)** omissions on reported mind wandering. Plots **(A,B)** use the data from the counterbalanced group from Model 1.1. Plot **(C)** uses the data from all groups from Model 1.7, and Plot **(D)** uses the data from all groups from Model 1.8.

Finally, we considered four relations previously documented in the literature. Focusing on the Counterbalanced group only, we examined whether the interval between probes (i.e., the number of trials preceding a thought probe) was related to reports of mind wandering. The probe intervals ranged from 5 to 101 trials (*M* = 49.55, *SD* = 22.33). Longer probe intervals (i.e., the number of trials preceding a thought probe) had previously been correlated with greater reports of overall mind wandering (Seli et al., 2013a). In Model 1.1 (Table 1) we distinguished between mind wandering subtypes and found that longer probe intervals were associated with increased reports of spontaneous mind wandering but that probe intervals did not have a substantiative impact on deliberate mind wandering (see Figure 3A). These findings suggest that while individuals can readily engage in deliberate mind wandering independently of the interval between thought probes, spontaneous mind wandering likely becomes more prominent during longer uninterrupted periods.

We then examined how spontaneous and deliberate mind wandering reports varied over time on task (i.e., the number of seconds spent performing the MRT). Focusing on the Counterbalanced group, Model 1.1 showed that collapsing across mind wandering instructions, over time mind wandering reports became *more* spontaneous and *less* deliberate. These patterns are shown in Figure 3B. The increase in spontaneous mind wandering over time on task is consistent with prior studies showing an increase in overall mind wandering with time on task in situations when people are asked to focus on the task at hand (Thomson et al., 2014). The decline in deliberate mind wandering over time on task might be particular to the present study in which participants were instructed to mind wander, perhaps reflecting the possibility that participants needed to spend progressively less effort on deliberately mind wandering to comply with our instructions—because spontaneous mind wandering was increasing and participants were aware of that and took account that it was happening in their metacognitive attempts to follow instructions. Alternatively, the temporal decline in deliberate mind wandering could reflect an increasing loss of cognitive control over time, making participants less able to deliberately mind wander as time on task increased, and allowing spontaneous mind wandering to creep in as cognitive control lessened.

Finally, across all groups (Counterbalanced, Ascending, and Descending), we examined whether the average rhythmic response time variability (RRTv) of the trials preceding a probe or the number of omission errors preceding a probe were related to participants’ reported levels of spontaneous and deliberate mind wandering. Model 1.7 (Table 1) analyzed the RRTv data, Model 1.8 (Table 1) analyzed the omission data, and the results are shown in Figures 3C,D respectively. The results showed that rhythmic response time variability and omissions increased with increases in both forms of mind wandering (supporting prior work; Anderson et al., 2021; Seli et al., 2013b). These results further support our assumption that changes in reported levels of mind wandering reflect changes in task attention and thus influence task performance.

## 3 Experiment 2

While collecting data for Experiment 1, we noticed some participants tended to shut their eyes or physically turn away from the computer monitor when instructed to mind wander during the task. Given that the MRT is an auditory task, and the computer screen was blank between probes, task performance did not require participants to view the monitor between probes. Yet keeping the eyes open during the task did provide the opportunity for visual distractions, which could co-opt attention resources from the task of mind-wandering, especially if the mind-wandering episode involved visual imagery. Thus, it seems possible that sensory detachment from the visual environment may aid participants in regulating their mind wandering without impeding the processing of the auditory task stimulus. Consistent with these considerations, many descriptions of mind wandering, like ours, often include an aspect of perceptual decoupling from the external task environment (Smallwood et al., 2008; Schooler et al., 2011; Kam and Handy, 2013; Baird et al., 2014). Prior studies have examined how various indices of gaze are associated with mind wandering (Faber et al., 2020), how activity in neural networks (e.g., the default mode network) depends on whether the eyes are open or closed (Patriat et al., 2013; Costumero et al., 2020), and how eye-blinking is related to mind wandering (Smilek et al., 2010).

Considering our informal observations, the primary aim of Experiment 2 was to examine whether participants’ control of their mind wandering was influenced by keeping their eyes open or closed. As in Experiment 1, participants were instructed to mind wander either 20, 40, 60, or 80% of the time across blocks of the MRT. We again measured both spontaneous and deliberate mind wandering using intermittent thought probes and we monitored performance by measuring response variability and omissions in the MRT. In addition to instructing participants to mind wander different amounts, participants were asked to alternate between keeping their eyes open or shut. The order of the instructions to mind wander different amounts and whether to keep the eyes open or shut were fully counterbalanced. If shutting one’s eyes helps to enable mind wandering, we would expect to see increased mind wandering reports and poorer performance in the MRT blocks when participants’ eyes are shut than when they are open. Furthermore, if shutting one’s eyes allows participants to better approximate the instructed levels of mind wandering, then we might expect mind wandering to better match instructed levels when the eyes are closed than when they are shut.

A secondary aim of Experiment 2 was to replicate the main findings from Experiment 1. In Experiment 2 we only included the situation in which the instructions to mind wander different amounts were counterbalanced across participants. We did not include the ascending and descending conditions of Experiment 1. Accordingly, we expect that as in Experiment 1, both spontaneous and deliberate mind wandering would increase with increases in instructed level of mind wandering, and that this effect should be larger for deliberate than spontaneous mind wandering. In addition, we again expected to see response variability and omission errors during the MRT increase with increases in instructed levels of mind wandering. Finally, we sought to replicate prior findings regarding how deliberate and spontaneous mind wandering changed as a function of probe interval and time on task, and how MRT performance prior to the probes varied as a function of deliberate and spontaneous mind wandering reports.

### 3.1 Methods

#### 3.1.1 Participants

Participants (*N* = 96) were recruited from the University of Waterloo undergraduate SONA research pool to participate in a 30-min study. The sample size of 96 was selected to allow each permutation of the 20, 40, 60, 80% instruction order to be fully counterbalanced with alternating instructions for participants to keep their eyes open or shut while completing blocks of the MRT. Three participants were replaced, two were removed due to non-compliance with instructions, another reported experiencing an adverse medical event during the task. The final sample consisted of 73 females, 19 males, and four participants who did not disclose their sex, with ages ranging from 17 to 30 (*M* = 19.64, *SD* = 2.07).

#### 3.1.2 Materials

The protocol for the MRT and Mind Wandering Thought Probes was identical to Experiment 1 apart from added instructions for participants to alternate having their eyes open or closed during the MRT. During the practice block of the MRT all participants had their eyes open to help familiarize them with the task. When responding to thought probes participants were instructed to report their mind wandering, then return to keeping their eyes open or shut as was instructed before continuing the block.

During the task whenever a thought probe appeared on the screen the tones from the MRT would pause while participants responded to the probe. If a participant’s eyes were closed the paused tone would signal to participants to open their eyes to respond to the probe. Inspection of video recordings of a subset of participants and researcher observation notes from the experiment indicate compliance with the eye condition instructions.

#### 3.1.3 Procedure

Data collection for Experiment 2 followed the same procedures as Experiment 1 with a few notable exceptions. In Experiment 2, there were two sets of instructions. At the start of each of the four blocks of the MRT participants were instructed (1) how much they should mind wander and (2) whether they should keep their eyes open or shut. The instructions to keep the eyes open or shut alternated across blocks and the starting state (open or shut) was counterbalanced across individuals such that each participant would complete half of the possible mind wandering and eye condition combinations. The order of mind wandering instructions was fully counterbalanced across participants and unlike Experiment 1 there were no groups receiving their mind wandering instructions in a special sequence (i.e., ascending or descending). An example sequence of blocks for a participant might be as follows: Block 1–20% mind wandering, eyes open; Block 2–40% mind wandering, eyes closed; Block 3–60% mind wandering, eyes open; Block 4–80% mind wandering, eyes closed). All 48 possible permutations of the mind wandering amounts and eye instructions were run twice.

Participants were run in cohorts of up to four at a time seated at computers in a large room oriented in different directions with separating dividers between them. Participants wore over-ear headphones while completing the task. All cohorts of participants began each block at the same time. At the end of each block participants waited for all members of their cohort to reach the same point before proceeding. At the start of each block the researcher approached participants individually to deliver their individual instructions for the following block.

### 3.2 Analysis

We used a series of independent theoretically informed models to conduct different statistical tests of our hypothesis. Model summaries including formulas, the observations examined, performance indicators, and ANOVAs for the predictors are available in Tables 3, 4.

**Table 3:** ANOVA tables for the linear mixed effects models examining mind wandering reports and response time variability for Experiment 2.

Model	Parameter	*SS*	*MS*	*df_Num_*	*df_Den_*	*F*	*p*
Model 2.1	Model Formula: MW ~ instruction*type*eye + probe_interval*type + time*type + (instruction | participant:type:eye) Model Data: Observations = 3,057, Performance: R^2^_Marginal_ = 0.217, R^2^_Conditional_ = 0.660						
	Instruction	58645.97	19548.66	3	236.3	76.783	<0.000
	Type	32502.75	32502.75	1	1282.0	127.663	<0.000
	Eye	575.69	575.69	1	375.9	2.261	0.133
	Probe_interval	33212.69	33212.69	1	2462.5	130.452	<0.000
	Time	403.17	403.17	1	550.3	1.584	0.209
	Instruction:type	15084.84	5028.28	3	236.3	19.750	<0.000
	Instruction:eye	2600.73	866.91	3	236.3	3.405	0.018
	Type:eye	71.74	71.74	1	375.9	0.282	0.596
	Type:probe_interval	15036.86	15036.86	1	2462.5	59.061	<0.000
	Type:time	23303.27	23303.27	1	550.3	91.530	<0.000
	Instruction:type:eye	2201.18	733.73	3	236.3	2.882	0.037
Model 2.2	Model Formula: RRTv ~ instruction*eye + (instruction | participant:eye) Model Data: Observations = 82,268, Performance: R^2^_Marginal_ = 0.004, R^2^_Conditional_ = 0.333						
	Instruction	13.87	4.62	3	93.9	3.459	0.019
	Eye	0.65	0.65	1	184.8	0.486	0.486
	Instruction:eye	1.13	0.38	3	93.9	0.282	0.838
Model 2.4	Model Formula: MW ~ RRTv*type + probe_interval*type + time*type + (1 | participant:type) Model Data: Observations = 3,043, Performance: R^2^_Marginal_ = 0.081, R^2^_Conditional_ = 0.319						
	RRTv	26880.364	26880.364	1	1994.653	54.244	<0.000
	Type	3528.628	3528.628	1	1869.274	7.121	0.008
	Probe_interval	37034.096	37034.096	1	2865.097	74.734	<0.000
	Time	1789.563	1789.563	1	2888.292	3.611	0.057
	RRTv:type	53.616	53.616	1	1994.653	0.108	0.742
	Type:probe_interval	18399.010	18399.010	1	2865.097	37.129	<0.000
	Type:time	52128.607	52128.607	1	2888.292	105.195	<0.000
Model 2.5	Model Formula: MW ~ omissions*type + probe_interval*type + time*type + (1 | participant:type) Model Data: Observations = 3,057, Performance: R^2^_Marginal_ = 0.059, R^2^_Conditional_ = 0.303						
	Omissions	6269.419	6269.419	1	3035.034	12.471	<0.000
	Type	41176.413	41176.413	1	749.893	81.908	<0.000
	Probe_interval	29587.659	29587.659	1	2871.449	58.856	<0.000
	Time	474.897	474.897	1	2873.056	0.945	0.331
	Omissions:type	1303.496	1303.496	1	3035.034	2.593	0.107
	Type:probe_interval	16534.099	16534.099	1	2871.449	32.890	<0.000
	Type:time	51908.117	51908.117	1	2873.056	103.256	<0.000

**Table 4:** ANOVA tables for the generalized mixed effects model examining omission errors for Experiment 2.

Model	Parameter	npar	*SS*	*MS*	*F*	*p*
Model 2.3	Model Formula: omissions ~ instruction*eye + (1 | participant:eye) Model Data: Observations = 82,944, Performance: R^2^_Marginal_ = 0.030, R^2^_Conditional_ = 0.558					
	Instruction	3	63.36	21.12	21.120	<0.000
	Eye	1	0.86	0.86	0.858	<0.000
	Instruction:eye	3	75.80	25.27	25.26	<0.000

The dependent measures included reports of spontaneous and deliberate mind wandering (Model 2.1, 2.4, 2.5), MRT RRTv (Model 2.2), and omissions (Model 2.3). These outcomes were analyzed using the lme4 package (Bates et al., 2015) in R version 4.3.3 (R Core Team, 2024) with linear mixed effects models (Models 2.1, 2.2, 2.4, 2.5) and a generalized mixed effects model using a logit link (Model 2.3). Model assumptions were checked using the performance package (Lüdecke et al., 2021). Post-hoc analyses were performed using the emmeans package with Tukey’s HSD to adjust for multiple pairwise comparisons (Lenth, 2023). The linear mixed effects model ANOVA results presented in Table 3 tested significance using the Satterthwaite approximation for degrees of freedom with the lmerTest package (Kuznetsova et al., 2017). The generalized mixed effects model ANOVA results presented in Table 4 used a Likelihood Ratio Test (LRT) to test the significance of the predictors.

Model 2.1 (Table 3) examined how our mind wandering instructions (“20,” “40,” “60” or “80”), and eye instructions (“open,” “shut”) influenced reports of mind wandering, while considering differences in the type of mind wandering report (“spontaneous” or “deliberate”). In the fixed effects we included time on task and probe interval as we found in Experiment 1 these factors help to explain variability in mind wandering reports. Our random effects specified random intercepts and slopes for each participant. Unique slopes and intercepts were considered for both types of mind wandering report, and whether participants had their eyes open or shut. This allowed the model to consider if there is an interaction between mind wandering instructions and eye instructions that varies across participants.

Model 2.2 (Table 3) examined how our mind wandering, and eye instructions influenced performance on the MRT as measured by RRTv. Here the fixed effects included our mind wandering and eye instructions and any interaction between them. The random effects again specify random slopes and intercepts, with unique slopes and intercepts considered for blocks where participants had their eyes open or shut.

Model 2.3 (Table 4) examined how our mind wandering, and eye instructions influenced the odds of omission errors in the MRT. Here the fixed effects included our mind wandering and eye instructions and any interaction between them. The random effects specified unique intercepts for blocks where participants had their eyes open or shut, only random intercepts were implemented to promote convergence of the generalized model.

Model 2.4 (Table 3) examined whether rhythmic response time variability scores predict subsequent mind wandering reports. We considered whether the relationship between RRTv and mind wandering reports differed for spontaneous and deliberate mind wandering. Time and probe interval were included as fixed effects to help explain variability in the outcomes. The random effects included random intercepts for each participant, separated by mind wandering subtype.

Model 2.5 (Table 3) examined whether missing responses predict subsequent mind wandering reports. We considered whether the relationship between omissions and mind wandering reports differed for spontaneous and deliberate mind wandering. Time and probe interval were included as fixed effects to help explain variability in the outcomes. The random effects included random intercepts for each participant, separated by mind wandering subtype.

### 3.3 Results and discussion

**Figure 4:**
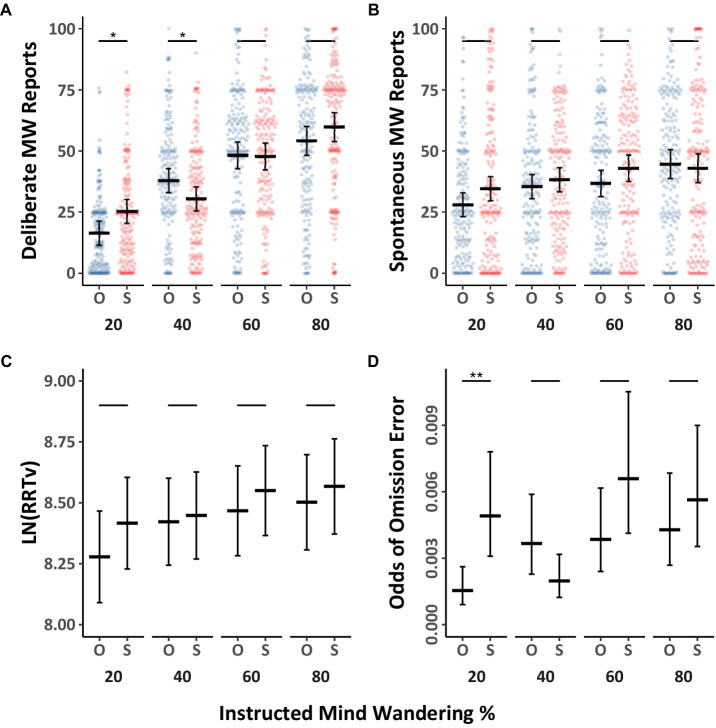
Differences in mind wandering outcomes and MRT performance for Experiment 2. Panels **(A,B)** contain mind wandering reports and task performance across levels of mind wandering instruction comparing individuals instructed to have their eyes opened “O” or shut “S.” The colored points illustrate raw participant responses to the mind wandering probes at every level of mind wandering instruction. The estimated marginal means, and 95% confidence intervals from Model 2.1 for panels **(A,B)**, Model 2.2 for panels **(C)**, and Model 2.3 for panel **(D)** are presented in black. Pairwise comparisons with significance scores are shown at the top, Tukey’s HSD was used to adjust for multiple comparisons (*p < 0.05, **p < 0.01, ***p < 0.001).

**Figure 5:**
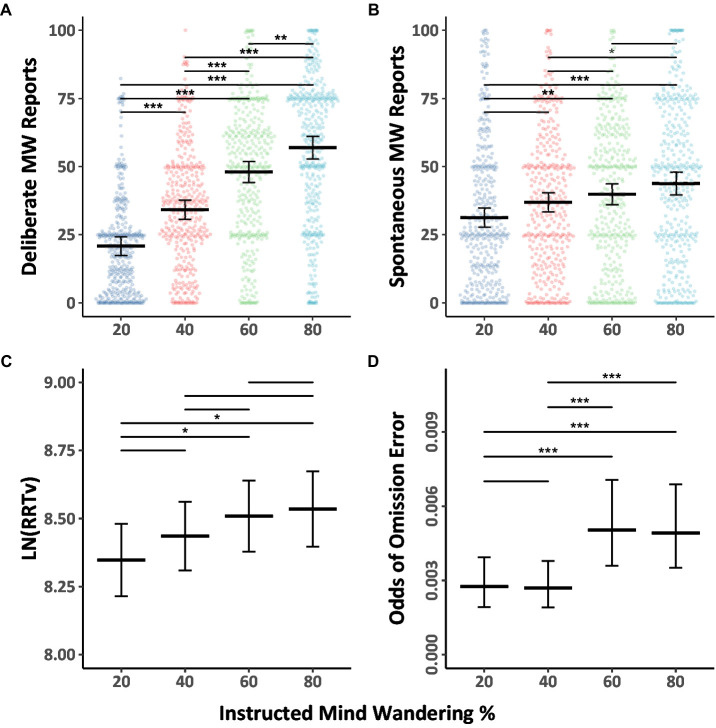
Differences in mind wandering outcomes and MRT performance for Experiment 2. The panels herein collapse the eye instructions to highlight the pairwise comparisons across levels of mind wandering instruction. The colored points illustrate raw participant responses to the mind wandering probes at every level of mind wandering instruction. The estimated marginal means, and 95% confidence intervals from Model 2.1 for panels **(A,B)**, Model 2.2 for panels **(C)**, and Model 2.3 for panel **(D)** are presented in black. Pairwise comparisons with significance scores are shown at the top, Tukey’s HSD was used to adjust for multiple comparisons (*p < 0.05, **p < 0.01, ***p < 0.001).

We first examined whether deliberate and spontaneous mind wandering were influenced by the eyes being open or shut, as well as instructions to mind wander various amounts. The mind wandering data were analyzed using Model 2.1 (Table 3), and they are depicted separately for eyes open, and eyes shut in Figures 4A,B (with Tukey HSD comparisons across eyes open/shut conditions), as well as collapsed across eyes open/shut conditions in Figures 5A,B (with Tukey HSD comparisons across levels of instructed mind wandering). Overall, we found weak evidence that shutting the eyes increases mind wandering. Paired comparisons between open and shut eye conditions at the four levels of instructed mind wandering revealed that when participants were instructed to mind wander 20% of the time, there was a modest increase in reports of deliberate mind wandering when eyes were closed compared to when they were open. However, the instructions to open or shut one’s eyes did not have a discernible impact on mind wandering reports at most other levels of instructed mind wandering. Contrary to our expectations, compared to keeping the eyes open, shutting the eyes resulted in a significant decrease in deliberate mind wandering at the 40% instructed level of mind wandering. Overall, we take the results of Experiment 2 to show that whether the eyes are closed or open had a negligible impact on mind wandering. Nevertheless, replicating the findings from Experiment 1 we found that both deliberate and spontaneous mind wandering increased with increases in instructed levels of mind wandering, with the effect being larger for deliberate compared to spontaneous mind wandering.

The response variability data and omission errors from the MRT were analyzed with Model 2.2 (Table 3) and 2.3 (Table 4) respectively. The results as a function of mind wandering instructions are presented in Figures 4C,D, which depicts data for eyes open and shut separately, and Figures 5C,D, which shows the data collapsed across the eyes open/shut conditions. The results showed no discernable influence of eye closure on RRTv performance. Omission errors follow a similar trend with deliberate mind wandering demonstrating a significant increase at the 20% level when the eyes are shut. The differences at all other levels of instruction are not significant. Consistent with Experiment 1, response variability and omission errors increased with instructions to mind wander more. Thus, Experiment 2 replicated our prior findings and additionally suggests that eye closure is not a critical factor in the present studies. However, it is worth noting that in the present experiments the probe intervals were all under 2.5 min in length. We did not assess whether closing one’s eyes can influence mind wandering during longer intervals.

**Figure 6:**
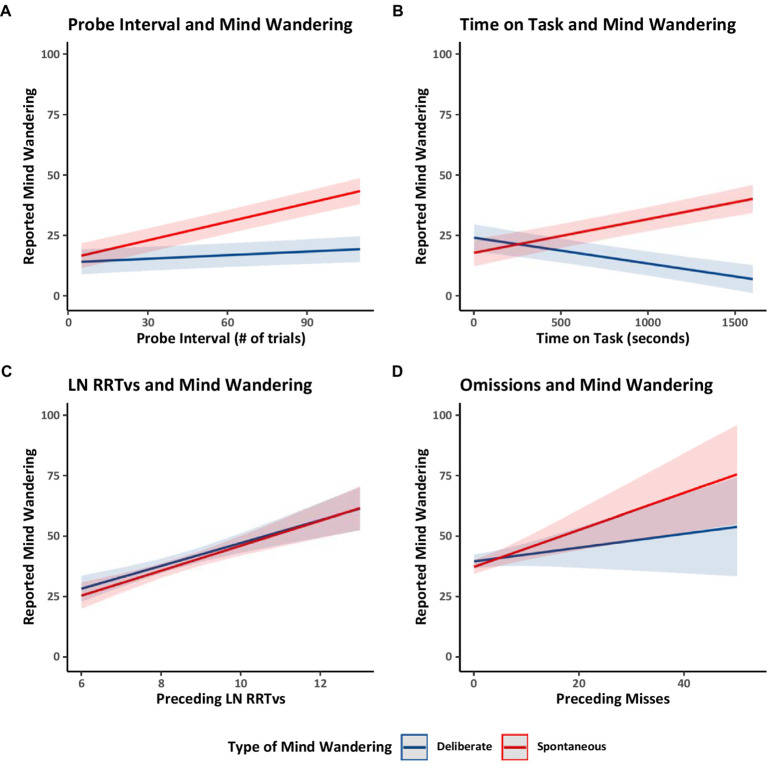
Estimated marginal trends in spontaneous and deliberate mind wandering reports for Experiment 2. Estimated marginal trends for the effects of **(A)** probe interval, **(B)** time on task, **(C)** rhythmic response time variability, and **(D)** omission on reported mind wandering. Plots **(A,B)** use the data from Model 2.1, while Plot **(C)** uses data from Model 2.4, and Plot **(D)** uses data from Model 2.5.

As in Experiment 1 we examined trends related to mind wandering reports and found highly similar patterns. In Model 2.1 (Table 3), the probe intervals ranged from 5 to 106 trials (*M* = 49.37, *SD* = 22.44). Longer intervals between probes were associated with greater reports of mind wandering, especially spontaneous mind wandering (Figure 6A). Again, in Model 2.1 (Table 3) more time on task was associated with more spontaneous and less deliberate mind wandering (Figure 6B). Increases in both deliberate and spontaneous mind wandering were related to poorer performance on trials just before the thought probes, as indicated in Model 2.4 (Table 3) by higher RRTv scores (Figure 6C) and in Model 2.5 (Table 3) by more omission errors (Figure 6D).

## 4 Experiment 3

In Experiment 3 we aimed to compare mind wandering outcomes and task performance between a group of participants instructed to mind wander different amounts (20, 40, 60, and 80% of the time as in Experiments 1 and 2) and a group of participants who were not so instructed and were simply allowed to mind wander naturally (i.e., a control group). Given that prior estimates from the extant literature suggest baseline mind wandering rates of between 30 and 50% (Killingsworth and Gilbert, 2010), we expected that participants receiving our instructions to mind wander 60% or 80% of the time would demonstrate mind wandering outcomes above those of the natural mind wandering control group. It is also possible that participants instructed to mind wander 20% of the time might be reducing their mind wandering relative to the control group despite being instructed to mind wander. Regarding task performance, the design of Experiment 3 also provided the opportunity to assess the possibility that controlling mind wandering to match an instructed amount might itself create a cognitive load that could impair task performance by taking limited resources from the metronome task. This would manifest in greater response time variability and more omissions in the instructed group compared to the control group, perhaps even when it comes to the lowest level (20%) of instructed mind wandering in the instructed group.

We also aimed to replicate the effects of mind wandering instructions shown in Experiments 1 and 2 using a slightly different mind wandering thought probe. Before beginning the MRT in Experiments 1 and 2, participants were told that when a mind wandering thought probe was presented, they should report how much they were mind wandering “right before the probe appeared” using a scale that ranged from “Not at All” to “All the Time.” After completing the experiments, we were concerned that the instructions “*right before*” could be interpreted in two ways. One interpretation is that “right before” refers to the period between the current probe and the prior probe (or the beginning of the block for the first probe). Another interpretation is that “right before” refers to the immediate instant before the probe’s appearance, in which case the response labels identifying to a span of time (e.g., “All the Time”) would be difficult to interpret. To address this lack of clarity in the thought probes, in Experiment 3 we changed the instructions regarding how participants should report their mind wandering. Specifically, we instructed participants to report on their experiences in the span of time between the current probe and the prior probe (or the beginning of the block for the first).

Finally, for the sake of completeness we again aimed to replicate and confirm several previous findings related to deliberate spontaneous mind wandering. Specifically, we explored how both spontaneous and deliberate mind wandering change as a function of thought probe interval and time on task. Furthermore, we again examined how response time variability and omissions during the MRT, measured on the trials before each thought probe, vary as a function of the amount of spontaneous and deliberate mind wandering reported by participants.

### 4.1 Methods

#### 4.1.1 Participants

Two groups of 48 participants (*N* = 96) were recruited from the University of Waterloo undergraduate SONA research pool to participate in a 30-min study. The group size of 48 was selected to allow each permutation of the 20, 40, 60, 80% mind wandering instruction order to be implemented twice for the group of participants receiving these instructions. The sample consisted of 73 Females and 23 Males, with ages ranging from 16 to 33 (*M* = 19.6, *SD* = 2.3).

#### 4.1.2 Materials

The procedure for the MRT, the mind wandering thought probes, and the mind wandering instructions were identical to those of Experiments 1 and 2. However, the instructions regarding the mind wandering thought probes at the beginning of the study were modified to enhance clarity of the probes. Specifically, participants were informed that at four points throughout each of the main blocks of the MRT they would be asked to report on their experiences of spontaneous and deliberate mind wandering. Participants were instructed that for the first thought probe in each block they should report on their experience of mind wandering from the start of the block to when that first probe appeared and that for subsequent thought probes they should report on their mind wandering from the last probe to the current one. Participants were told that each of their mind wandering reports should be for distinct non-overlapping periods of time.

#### 4.1.3 Procedure

Participants were randomly assigned to receive instructions to mind wander specific amounts (the Instructed group) or to not receive instructions to mind wander and allow mind wandering to occur naturally (the Control group). Participants in both groups were informed that some participants would receive instructions to mind wander either 20, 40, 60, or 80% of the time at the start of each block. In the Instructed group the order of the instructions was counterbalanced across individuals. In the Control group participants did not receive instructions on their level of mind wandering, at the start of each block they were instructed to proceed when ready. Data collection for this experiment was done with cohorts of up to four participants at a time following the same protocol described in Experiment 2. Cohorts of the Instructed and Control groups were run separately.

### 4.2 Analysis

We used a series of independent theoretically informed models to conduct different statistical tests of our hypothesis. Model summaries including formulas, the observations examined, performance indicators, and ANOVAs for the predictors are available in Tables 5, 6.

**Table 5:** ANOVA tables for the linear mixed effects models examining mind wandering reports and response time variability for Experiment 3.

Model	Parameter	*SS*	*MS*	*df_Num_*	*df_Den_*	*F*	*p*
Model 3.1	Model Formula: MW ~ instruction*type + probe_interval*type + time*type + (instruction | participant:type) Model Data: Instructed Group, Observations = 1,535, Performance: R^2^_Marginal_ = 0.249, R^2^_Conditional_ = 0.718						
	Instruction	31777.81	10592.60	3	92.5	51.701	<0.000
	Type	7534.96	7534.96	1	235.8	36.777	<0.000
	Probe_interval	11083.82	11083.82	1	1231.3	54.099	<0.000
	Time	87.06	87.06	1	265.4	0.425	0.515
	Instruction:type	3825.94	1275.31	3	92.5	6.225	0.001
	Type:probe_interval	7682.22	7682.22	1	1231.3	37.496	<0.000
	Type:time	6432.79	6432.79	1	265.4	31.398	<0.000
Model 3.2	Model Formula: RRTv ~ instruction + (instruction | participant) Model Data: Instructed Group, Observations = 41,051, Performance: R^2^_Marginal_ = 0.004, R^2^_Conditional_ = 0.313						
	Instruction	13.75	4.58	3	46.9	3.409	0.025
Model 3.4	Model Formula: MW ~ instruction*type + probe_interval*type + time*type + (1 | participant:type) Model Data: All Groups, Observations = 3,061, Performance: R^2^_Marginal_ = 0.171, R^2^_Conditional_ = 0.551						
	Instruction	211242.59	52810.65	4	957.5	155.002	<0.000
	Type	5446.13	5446.13	1	344.1	15.985	<0.000
	Probe_interval	42101.79	42101.79	1	2861.2	123.571	<0.000
	Time	21025.37	21025.37	1	2862.0	61.711	<0.000
	Instruction:type	28532.27	7133.07	4	957.5	20.936	<0.000
	Type:probe_interval	10236.18	10236.18	1	2861.2	30.044	<0.000
	Type:time	10735.33	10735.33	1	2862.0	31.509	<0.000
Model 3.5	Model Formula: RRTv ~ instruction + (1 | participant) Model Data: All Groups, Observations = 82,519, Performance: R^2^_Marginal_ = 0.016, R^2^_Conditional_ = 0.253						
	Instruction	338.31	84.58	4	368.7	64.702	<0.000
Model 3.7	Model Formula: MW ~ RRTv*type + probe_interval*type + time*type + (1 | participant:type) Model Data: All Groups, Observations = 3,051, Performance: R^2^_Marginal_ = 0.095, R^2^_Conditional_ = 0.404						
	RRTv	18261.43	18261.43	1	2748.5	44.159	<0.000
	Type	1828.18	1828.18	1	2483.8	4.421	0.036
	Probe_interval	39441.54	39441.54	1	2856.6	95.375	<0.000
	Time	12444.64	12444.64	1	2896.9	30.093	<0.000
	RRTv:type	3705.83	3705.83	1	2748.5	8.961	0.003
	Type:probe_interval	9074.10	9074.10	1	2856.6	21.942	<0.000
	Type:time	12517.61	12517.61	1	2896.9	30.269	<0.000
Model 3.8	Model Formula: MW ~ omissions*type + probe_interval*type + time*type + (1 | participant:type) Model Data: All Groups, Observations = 3,061, Performance: R^2^_Marginal_ = 0.051, R^2^_Conditional_ = 0.439						
	Omissions	2029.86	2029.86	1	3038.8	4.811	0.028
	Type	4259.19	4259.19	1	469.1	10.095	0.002
	Probe_interval	36749.73	36749.73	1	2873.0	87.099	<0.000
	Time	18209.61	18209.61	1	2873.0	43.158	<0.000
	Omissions:type	1408.00	1408.00	1	3038.8	3.337	0.068
	Type:probe_interval	11595.83	11595.83	1	2873.0	27.483	<0.000
	Type:time	11731.83	11731.83	1	2873.0	27.805	<0.000

**Table 6:** ANOVA tables for the generalized mixed effects models examining omission errors for Experiment 3.

Model	Parameter	npar	*SS*	*MS*	*F*	*p*
Model 3.3	Model Formula: omissions ~ instruction + (instruction | participant) Model Data: Instructed Group, Observations = 41,472, Performance: R^2^_Marginal_ = 0.002, R^2^_Conditional_ = 0.400					
	Instruction	3	7.25	2.42	2.418	0.065
Model 3.6	Model Formula: omissions ~ instruction*group + (instruction | participant) Model Data: All Groups, Observations = 82,944, Performance: R^2^_Marginal_ = 0.047, R^2^_Conditional_ = 0.453					
	Instruction	4	16.75	4.19	4.188	0.003

The dependent measures included reports of spontaneous and deliberate mind wandering (Models 3.1, 3.4, 3.7, 3.8), MRT RRTv (Models 3.2, 3.5), and omission errors (Models 3.3, 3.6). These outcomes were analyzed using the lme4 package (Bates et al., 2015) in R version 4.3.3 (R Core Team, 2024) with linear mixed effects models (Models 3.1, 3.2, 3.4, 3.5, 3.7, 3.8) and generalized mixed effects models using a logit link (Models 3.3, 3.6). Model assumptions were checked using the performance package (Lüdecke et al., 2021). Post-hoc analyses were performed using the emmeans package with Tukey’s HSD to adjust for multiple pairwise comparisons (Lenth, 2023). The linear mixed effects model ANOVA results presented in Table 5 tested significance using the Satterthwaite approximation for degrees of freedom with the lmerTest package (Kuznetsova et al., 2017). The generalized mixed effects model ANOVA results presented in Table 6 used a Likelihood Ratio Test (LRT) to test the significance of the predictors.

Model 3.1 (Table 5) examined how our mind wandering instructions (“20,” “40,” “60” or “80”) influenced reports of mind wandering in the Instructed group, considering differences in the type of mind wandering report (“spontaneous” or “deliberate”). Here we replicated the modeling approach taken in Model 1.1 from Experiment 1. The fixed effects included time on task and probe interval. Our random effects specified random intercepts and slopes for each participant.

Model 3.2 (Table 5) assessed how the mind wandering instructions influenced RRTv performance on the MRT in the Instructed group. Here we replicated the modeling approach taken in Model 1.2 from Experiment 1. The fixed effects examined our instructions as the predictor with random slopes for each participant.

Model 3.3 (Table 6) assessed how the mind wandering instructions influenced omission errors on the MRT in the Instructed group. Here we replicated the modeling approach taken in Model 1.3 from Experiment 1. The fixed effects examined our instructions as the predictor with random intercepts for each participant.

Model 3.4 (Table 5) examined how our mind wandering instructions (“20,” “40,” “60,” “80” or “control”) influenced reports of mind wandering in both the Instructed and Control group, allowing us to make comparisons between the Instruction and Control groups broadly, or to compare mind wandering reports at specific levels of instruction with reports in the Control group. The fixed effects again included time on task and probe interval. Our random effects specified random intercepts for each type of mind wandering report. It was not possible to calculate random slopes for the instructions as participants in the Control group did not receive different instructions.

Model 3.5 (Table 5) examined how our mind wandering instructions influenced RRTv performance on the MRT in both the Instructed and Control group, allowing us to make comparisons between the Instruction and Control groups broadly, or to compare performance at specific levels of instruction with performance in the Control group. The fixed effects examined our instructions as the predictor. The random effects specified random intercepts.

Model 3.6 (Table 6) examined how our mind wandering instructions influenced omission errors on the MRT in both the Instructed and Control group, allowing us to make comparisons between the Instruction and Control groups broadly, or to compare performance at specific levels of instruction with performance in the Control group. The fixed effects examined our instructions as the predictor. The random effects specified random intercepts.

Model 3.7 (Table 5) examined whether rhythmic response time variability scores predict subsequent mind wandering reports. We considered whether the relationship between RRTv and mind wandering reports differed for spontaneous and deliberate mind wandering. Time and probe interval were included as fixed effects to help explain variability in the outcomes. The random effects included random intercepts for each participant, separated by mind wandering subtype.

Model 3.8 (Table 5) examined whether missing responses predict subsequent mind wandering reports. We considered whether the relationship between omissions and mind wandering reports differed for spontaneous and deliberate mind wandering. Time and probe interval were included as fixed effects to help explain variability in the outcomes. The random effects included random intercepts for each participant, separated by mind wandering subtype.

### 4.3 Results and discussion

**Figure 7:**
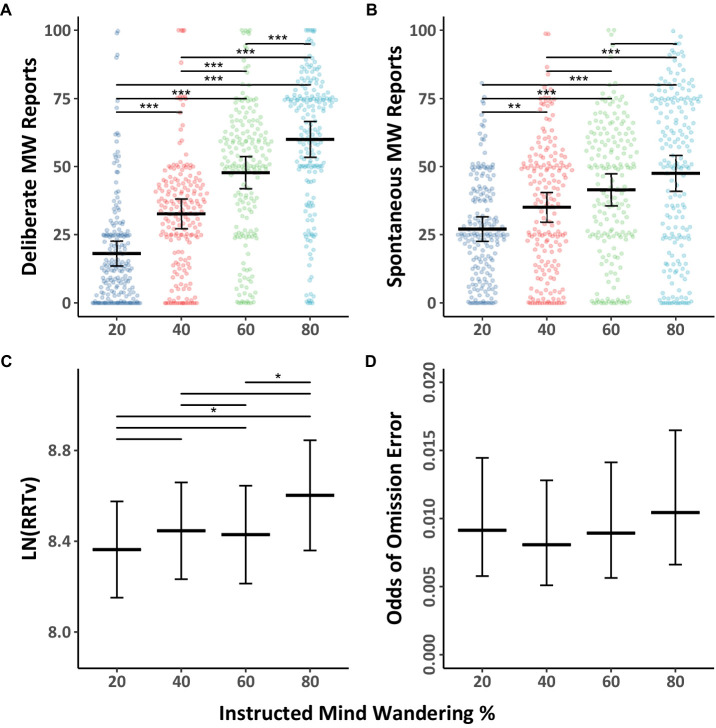
Mind wandering outcomes and MRT performance by instructed levels of mind wandering for Experiment 3. The colored points illustrate raw participant responses to the mind wandering probes at every level of mind wandering instruction. The estimated marginal means, and 95% confidence intervals from Model 3.1 for panels **(A,B)**, Model 3.2 for panel **(C)**, and Model 3.3 for panel **(D)** are presented in black. Pairwise comparisons with significance scores are shown at the top, Tukey’s HSD was used to adjust for multiple comparisons (*p < 0.05, **p < 0.01, ***p < 0.001).

We first replicated the pattern of evidence from our overarching hypothesis that participants can adjust their levels of mind wandering in response to instructions. The mind wandering probe data and performance data from the instructed group are depicted in Figure 7. These data were analyzed using Models 3.1 and 3.2 (Table 5), and Model 3.3 (Table 6). We observed the same pattern as in prior experiments whereby participants increased their mind wandering along with their response variability and proportion of omission errors when they were instructed to mind wander in increasing amounts. As before, deliberate mind wandering showed a much larger response to our instructions than did spontaneous mind wandering, leading to an interaction between instructed level of mind wandering and mind wandering subtype (see Model 3.1). Back transformation of the RRTv scores reveals when participants were instructed to mind wander 80% of the time the variability in their response times was >27% when they were instructed to mind wander 20% the time. While participants’ odds of making omission errors were 15% greater when instructed to mind wander 80% of the time relative to 20% of the time, the differences in omission scores were close to but did not reach significance. The apparently smaller effect of instructed mind wandering amounts on performance could be due to the smaller sample size in the analysis in this model relative to our prior experiments.

**Figure 8:**
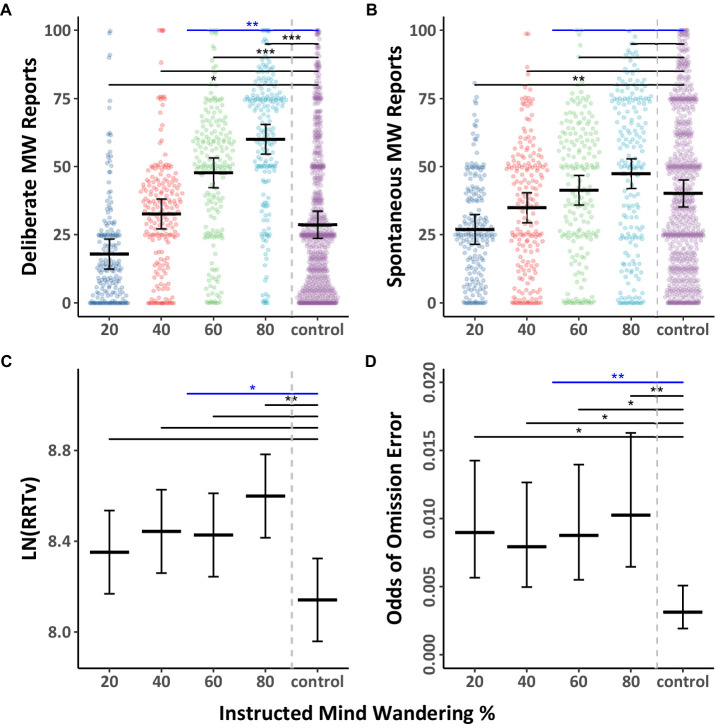
Mind wandering outcomes and MRT performance by instructed levels of mind wandering for Experiment 3. The colored points illustrate raw participant responses to the mind wandering probes at every level of mind wandering instruction. The estimated marginal means, and 95% confidence intervals from Model 3.4 for panels **(A,B)**, Model 3.5 for panel **(C)**, and Model 3.6 for panel **(D)** are presented in black. Pairwise comparisons with significance scores are shown at the top, with black lines indicating comparisons between specific levels of instruction and the control group, and blue lines indicating comparisons between the instruction and control group overall, Tukey’s HSD was used to adjust for multiple comparisons (*p < 0.05, **p < 0.01, ***p < 0.001).

We then compared the two groups’ participants to examine differences between instructed levels of mind wandering (the instructed group) and the absence of specific mind wandering instructions (the control group). The mind wandering probe data and performance data from both groups were analyzed using Models 3.4–3.6. The results from the models are shown in Tables 5, 6, though more important are the Tukey pairwise comparisons that were generated from the models, which are shown in Figure 8. As predicted, participants instructed to mind wander 60 or 80% of the time (levels thought to be higher than normal) had significantly higher deliberate mind wandering reports relative to the average of the control group (Figure 8A). Participants instructed to mind wander 20% of the time displayed significantly lower spontaneous mind wandering than those not given instructions to mind wander a specific amount (Figure 8B). In terms of behavior, participants asked to mind wander 80% of the time also displayed significantly higher RRTv than the control group (Figure 8C). Furthermore, across all levels of mind wandering instruction participants had significantly higher odds of making omission errors than those in the control group (Figure 8D). These results are consistent with the notion that instructions to mind wander create a cognitive load that impairs performance on the MRT.

**Figure 9:**
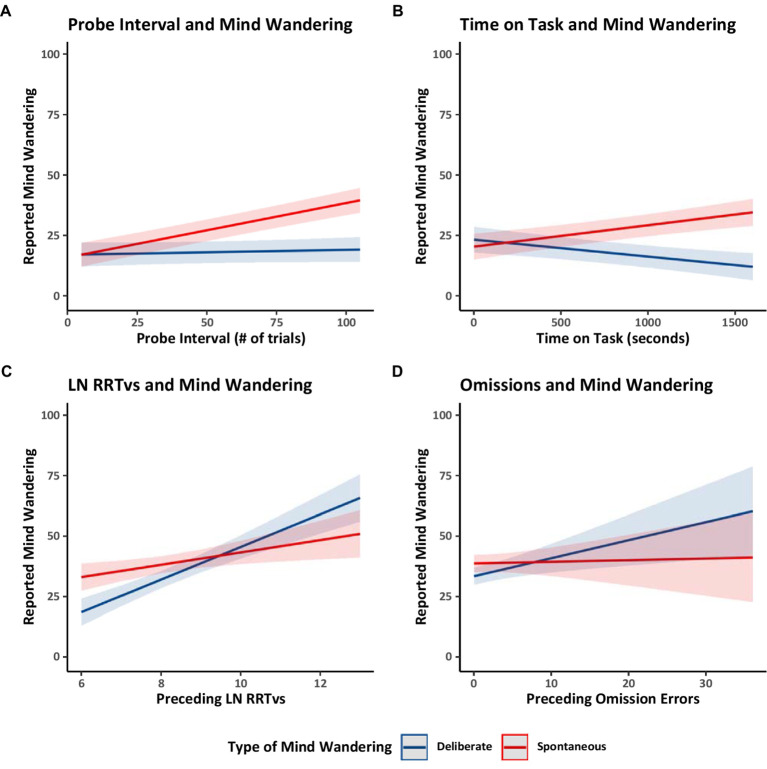
Estimated marginal trends in spontaneous and deliberate mind wandering reports for Experiment 3. Estimated marginal trends for the effects of **(A)** probe interval, **(B)** time on task, **(C)** rhythmic response time variability, and **(D)** omission on reported mind wandering. Plots **(A,B)** use the data from Model 3.1, while Plot **(C)** uses data from Model 3.7, and Plot **(D)** uses data from Model 3.8.

Examining the trends related to mind wandering reports we again found highly similar patterns to those shown in our previous studies. In Model 3.1, which examines the Instructed group (Table 5), the probe intervals ranged from 5 to 104 trials averaging (*M* = 49.31, *SD* = 22.50). Longer intervals between probes were again associated with greater reports of mind wandering, especially spontaneous mind wandering (Figure 9A). In Model 3.1 (Table 5), as before, more time on task was associated with more spontaneous and less deliberate mind wandering (Figure 9B). In both the Instructed and Control groups poorer performance on the set of trials preceding the thought probes was once again associated with higher reports of both spontaneous and deliberate mind wandering, as indicated in Model 3.7 (Table 5) by higher RRTv scores (Figure 9C) and in Model 3.8 (Table 6) by more omission errors (Figure 9D).

## 5 General discussion

In three experiments we showed that participants’ levels of self-reported mind wandering systematically varied in response to instructions to mind wander either 20, 40, 60, or 80% of the time while completing a sustained attention task (the MRT). As instructed levels of mind wandering increased, participants reported progressively more deliberate and spontaneous mind wandering; these instruction-related changes were consistently smaller for spontaneous mind wandering than for deliberate mind wandering. We also found that overall levels of mind wandering and the ability to modulate levels of mind wandering based on instructions were not strongly affected by whether participants’ eyes were open or closed. Instructions to mind wander 60 or 80% of the time led to higher levels of mind wandering than was reported by participants who were not instructed to mind wander specific amounts. In contrast, instructions to mind wander 20% of the time led to less spontaneous mind wandering than reported by individuals who were not given instructions to mind wander. Finally, across all experiments participants showed progressively poorer performance in the sustained attention task with increases in levels of instructed mind wandering.

Overall, these results are consistent with the conclusion that people can effectively modulate their levels of mind wandering on command. The degree of control over the amount of mind wandering is impressive, with participants on average being able to shift their mind wandering levels from roughly 20 to 60% of the time. However, participants consistently failed to match higher levels of instructed mind wandering (e.g., mind wandering 80% of the time), generally mind wandering less than instructed in such cases. It could be that people are able to mind wander to a high degree as instructed, but they prevent themselves from doing so to maintain a reasonable level of performance on the primary task. Controlling mind wandering to match an instructed amount appears to come at a cost in terms of performance, suggesting that controlling the allocation of resources to achieve specific amounts of mind wandering imposes a cognitive load and is resource demanding. Our findings are consistent with prior literature demonstrating instruction-based control over attentional deployment in both the visual (Sperling and Melchner, 1978) and auditory (Moray, 1959) domains.

One possible criticism of the present findings is that participants were not in fact adjusting their levels of mind wandering to match instructed amounts, but rather simply reporting what they perceived to be the expected or desired level of mind wandering. This alternative explanation would suggest the instruction-based changes in mind wandering were the result of response bias (i.e., a form of demand characteristic; see Nichols and Maner, 2008). While it is impossible to completely rule this out, there are several aspects of our findings that are inconsistent with (or at least inconvenient for) this alternative. First, we found replicable increments in response time variability and omissions—metrics that participants likely do not intuitively link to levels of mind wandering—that co-occurred with observed increases in instruction-based levels of mind wandering. In other words, the mind wandering instructions not only influenced subjective reports, but also task performance in a consistent way. Second, there were subtle patterns in the mind wandering responses that are unlikely to have been manufactured by participants. Examples include (1) that the mind wandering instructions impacted both spontaneous and deliberate mind wandering but to a lesser extent spontaneous mind wandering, (2) that spontaneous but not deliberate mind wandering increased with increases in probe interval, (3) that spontaneous mind wandering increased over time on task while deliberate mind wandering decreased over time, and (4) that overall, response variability and omissions in the trials preceding a thought probe increased with higher reported levels of mind wandering. Finally, we found poorer performance on the primary task when participants were instructed to mind wander than when they were not so instructed (Experiment 3), indicating that instructed participants were engaged in a mental activity that required attentional resources (i.e., likely controlling their mind wandering to match instructed levels). When all these aspects of the data are considered together as a whole, our findings are not easily explained by the notion that the instruction-related changes in mind wandering were simply the result of demand characteristics influencing reports in response to the occurrences of probes.

Our technique of asking participants to mind wander a certain amount has several advantages. First, the technique can be used to manipulate mind wandering while holding constant other factors, including participants’ levels of motivation (Seli et al., 2019), individuals’ current concerns (Stawarczyk et al., 2013), and various task and stimulus parameters typically varied to influence mind wandering, such as task difficulty (Seli et al., 2018c), interestingness (Unsworth and McMillan, 2013) and time on task (Thomson et al., 2014). Second, the technique may provide a novel avenue for exploring the role of cognitive control mechanisms in the direction of internal thoughts. Future research could, for example, examine how various substances that are thought to impair cognitive control and increase mind wandering—such as alcohol (Sayette et al., 2009) and cannabis (Adam et al., 2020)—influence the extent to which people can modulate their levels of deliberate and spontaneous mind wandering on command. Along similar lines, it may be worth exploring how instruction-related changes in mind wandering might vary as a function of control-related individual traits, such as working memory capacity (Rummel and Boywitt, 2014), and tendencies to experience attention lapses in everyday life (Cheyne et al., 2006). It could be that individuals who have lower working memory capacity or experience more attention lapses in everyday life will be less able to systematically vary their levels of mind wandering on command.

It is worth considering the extent to which our findings may depend on the difficulty of the primary task that we used. Because there are no critical stimuli in the MRT to which participants must respond in a unique way, the mental demands of the task are quite low compared to other vigilance tasks. The simplicity of the task provides participants considerable opportunity to engage in mind-wandering while simultaneously maintaining a reasonable level of performance. However, even in the present experiments participants’ mind wandering reports typically fell short of the instructed amounts when they were instructed to mind wander at higher levels (i.e., 60% or 80% of the time). This suggests that there is a considerable meta-cognitive demand being imposed by the instructions to mind-wander at these higher levels, which participants have difficulty matching, even when other task demands are low. Considering that the regulation of mind-wandering appears to impose additional mental load, participants may find it even harder to regulate their experiences of mind-wandering while simultaneously performing more difficult tasks. Furthermore, as primary tasks become harder, participants may struggle more to attain high levels of instructed mind-wandering without experiencing what to them might be unacceptably poor performance in the primary task.

The present studies also provided the opportunity to observe several mind wandering-related patterns reported in prior studies. First, in all of our studies we found that as previously reported (Seli et al., 2013a), mind wandering increased with increases in the temporal interval between successive mind wandering thought probes. This is consistent with the notion that frequent probes bring thoughts back to the task and reduce mind wandering. It is also consistent with the idea that controlling mind wandering requires effort and the exercise of that effort can dissipate over time. Second, in every study we replicated the general pattern that spontaneous mind wandering increases over time on task. However, we also found that deliberate mind wandering decreased over time on task, a finding that could be specific to this context in which participants were instructed to mind wander, and again consistent with the idea that deliberate mind wandering is an effortful task in which control can dissipate, deteriorate, or disappear.

Finally, the present findings are consistent with the notion that mind wandering is not simply a mental state that occurs unexpectedly because of a failure of control (McVay and Kane, 2010), but rather that people do sometimes mind wander intentionally and may be able to titrate their mind wandering levels strategically (Seli et al., 2018a). Prior research has demonstrated that individuals are capable of rapidly modulating their mind wandering in response to contextual demands, increasing their mind wandering when demands are expected to be lower and decreasing mind wandering in anticipation of increases in task demands (Seli et al., 2018a). The dynamic regulation of mind wandering can be understood adaptively as a means to enjoy the benefits of mind wandering while limiting any potential costs (Smallwood and Andrews-Hanna, 2013). By recognizing the agency that individuals have in managing their mental states we open avenues for further research into the mechanisms and strategies by which individuals direct their attention both toward and away from their current activities.

## Data Availability

The datasets presented in this study can be found in online repositories. The names of the repository/repositories and accession number(s) can be found in the article/supplementary material.
